# ASSOCIATION OF SOCIOECONOMIC STATUS, SOCIAL ACTIVITIES, AND LONELINESS WITH DEPRESSION AMONG OLDER ADULTS

**DOI:** 10.1093/geroni/igae098.2867

**Published:** 2024-12-31

**Authors:** Yaping Wang, Jue Liu

**Affiliations:** Peking University, Beijing, Beijing, China (People’s Republic); Peking University, Beijing, Beijing, China (People’s Republic)

## Abstract

To estimate whether social activities and loneliness mediate the association between SES and depression, and the extent of interactive or joint relations of SES, social activities, and loneliness with depression, we did a multi-cohort study utilizing five aging surveys across 24 countries. SES was defined as high- and low-level by latent class analysis based on family income, education, and work status. Depression was assessed by 8-item or 10-item Center for Epidemiological Studies Depression Scale (CES-D) and EURO-D. Cox proportional hazard models were applied to estimate the association of SES with depression. Random effects models were used to pool results. A total of 79638 participants were included in our study. With a median follow-up of 5-year, compared with participants in high-level SES, those in low-level SES had a pooled 29.1% (pooled Hazard Ratio [pHR] =1.291, 95%CI: 1.188-1.393) higher risk of depression after adjusting all covariates, and the pooled proportions mediated by social activities and loneliness were 8.1% (95%CI: 3.2-12.9%) and 5.8% (95%CI: 3.6-7.9%), respectively. No significant additive interactions were observed between SES and social activities or loneliness. Compared with participants in high-level SES, socially-active, and not lonely group, those in low-level SES, socially-inactive, and lonely group had a 133.5% increased risk of depression (pHR=2.335, 95%CI: 2.058-2.612). Social activities and loneliness mediated a small proportion of socioeconomic inequality in depression, which indicated that other pathways addressing socioeconomic inequality were required. Additionally, the joint effects of SES, social activities, and loneliness highlighted the benefits of simultaneous and integrated interventions to reduce depression burden.

